# Action Recognition Using Single-Pixel Time-of-Flight Detection

**DOI:** 10.3390/e21040414

**Published:** 2019-04-18

**Authors:** Ikechukwu Ofodile, Ahmed Helmi, Albert Clapés, Egils Avots, Kerttu Maria Peensoo, Sandhra-Mirella Valdma, Andreas Valdmann, Heli Valtna-Lukner, Sergey Omelkov, Sergio Escalera, Cagri Ozcinar, Gholamreza Anbarjafari

**Affiliations:** 1 iCv Lab, Institute of Technology, University of Tartu, 50411 Tartu, Estonia; 2 University of Barcelona, 08007 Barcelona, Spain; 3 Institute of Physics, University of Tartu, 50411 Tartu, Estonia; 4 The Computer Vision Centre, 08193 Barcelona, Spain; 5 Trinity College Dublin, Dublin 2, Ireland; 6 Department of Electrical and Electronic Engineering, Hasan Kalyoncu University, Gaziantep 27000, Turkey; 7 Institute of Digital Technologies, Loughborough University London, London E15 2GZ, UK

**Keywords:** single pixel single photon image acquisition, time-of-flight, action recognition

## Abstract

Action recognition is a challenging task that plays an important role in many robotic systems, which highly depend on visual input feeds. However, due to privacy concerns, it is important to find a method which can recognise actions without using visual feed. In this paper, we propose a concept for detecting actions while preserving the test subject’s privacy. Our proposed method relies only on recording the temporal evolution of light pulses scattered back from the scene. Such data trace to record one action contains a sequence of one-dimensional arrays of voltage values acquired by a single-pixel detector at 1 GHz repetition rate. Information about both the distance to the object and its shape are embedded in the traces. We apply machine learning in the form of recurrent neural networks for data analysis and demonstrate successful action recognition. The experimental results show that our proposed method could achieve on average 96.47% accuracy on the actions walking forward, walking backwards, sitting down, standing up and waving hand, using recurrent neural network.

## 1. Introduction

Action is a spatiotemporal sequence of patterns [1,2,3,4,5,6]. The ability to detect movement and recognise human actions and gestures would enable advanced human to machine interaction in wide scope of novel applications in the field of robotics from autonomous vehicles, surveillance for security or care-taking to entertainment.

In the field of machine vision, the majority of effort has been put into recognising human action from video sequences [7,8,9], because overwhelmingly imaging devices mimic the human-like perception of the surroundings and video format is most widely available. Videos are a sequence of two-dimensional intensity patters, captured by using an imaging lens projecting the scene to a two-dimensional detector array (a charge coupled device (CCD) device, for example). Unlike living creatures, the ever growing field of robotics has run into major difficulties while trying to recognise objects, their actions, and their distances from two-dimensional images. Processing the data is computationally demanding, and depth information is not unanimously retrievable.

Deep neural networks, due to their high accuracy, are widely used in many of the computer vision applications such as emotion recognition [10,11,12,13,14,15,16], biometric recognition [17,18,19,20], personality analysis [21,22], and activity analysis [5,23,24]. Depending on the nature of the data, different structures can be used [25,26]. In this work, we deal with time-series data, i.e., we handle temporal information. For this purpose, we are mainly focused on recurrent neural networks (RNN) and long-short term (LSTM) algorithms.

In addition to colour and intensity, incident light can be characterised by its propagation direction(s), spectral content and temporal evolution in the case of pulsed illumination. Light also carries information about its source, and each medium, refraction, reflection and scattering event it has encountered or traversed. This enables various uncommon ways to characterise the scene. The rapid advancements in optoelectronics and availability of sufficient computational power enable innovative imaging and light capturing concepts, which serve as the ground for action detection. For example, a detector, capable of registering evolution of backscattered light with a high temporal resolution in a wide dynamic range, would be able to detect even objects hidden from the direct line of sight [27,28,29]. Along the same vein, several alternative light-based methods have been developed for resolving depth information or 3D map of the surroundings (some examples can be found in [30,31,32,33,34]) giving 3D information in voxel format about the scene, which is also suitable for action detection [35]. Combining the fundamental understanding of light propagation and computational neural networks for the data reconstruction, it appears that objects or even persons can be detected using a single pixel detector registering temporal evolution of the back-scattered light pulse [36].

In this work, which is a feasibility study of a novel setup and methodology for conducting action recognition, we propose and demonstrate an action recognition scheme based on a single-pixel direct time-of-flight detection. We use NAO robots in a controlled environment as a test subject. We illuminate a scene with a diverging picosecond laser pulse, (30 ps duration) and detect the temporal evolution of back scattered light with a single pixel multiphoton detector of 600 ps temporal resolution. Our data contains one-dimensional time sequences presenting the signal strength (proportional to the number of detected photons) versus arrival time. Information about both the distance to the object and its shape are embedded in the traces. We apply machine learning in the form of recurrent neural networks for data analysis and demonstrate successful action recognition.

The following list summarises the contributions of our work:

- Introduce an unexplored data modality for action recognition scenarios. In contrast to other depth-based modalities, our single-pixel light pulses are not visually interpretable which makes it a more privacy preserving solution.
- Provide a manually annotated dataset of 550 robot action sequences, some of them containing obstacle objects.
- Apply multi-layer bi-directional recurrent neural networks for the recognition task as an initial machine learning benchmarking baseline.
- Present an extensive set of experiments on the recognition of several action classes. We demonstrate how the learning models are able to extract proper action features and generalise several action concepts from captured data.

The rest of the paper is organised as follows: in Section 2, related works to single-pixel, single-photon acquisition and action recognition are reviewed. Section 3 describes the data collection and the details of the setup. In Section 4, the details of the proposed deep neural network algorithm used for action recognition are described. The experimental results and discussions are provided in Section 5. Finally, the work is concluded in Section 6.

## 2. Related Work

The filed of motion analysis was firstly inspired by intensity images and progresses towards depth images, which are more robust in comparison to intensity images. In the case of action recognition, the most useful are sensors that provide depth map. Nevertheless, data are processed to extract human silhouettes, body parts, skeleton and pose of the person, which in turn are used as features for machine learning methods to classify actions. These sensors have drawn much interest for human activity related research and software development.

### 2.1. Depth Sensors

Depth images provide the 3D structure of the scene, and can significantly simplify tasks such as background subtraction, segmentation, and motion estimation. With the recent advances in depth sensor hardware, such as time-of-flight (ToF) cameras, research based on depth imagery has appeared. Three main depth sensing technologies are applied in computer vision research: stereo cameras, time-of-flight (ToF) cameras and structured light.

1. Stereo cameras infer the 3D structure of a scene from two images from different viewpoints. The depth map is created using information about the camera setup (stereo triangulation) [37].
2. A time-of-flight (ToF) camera estimates distance to an object surface using active light pulses from a single camera, whose time to reflect from the object give the distance. Such devices use a sinusoidally modulated infra-red light signal, and distance is estimated using the phase shift of the reflected signal on CMOS or CCD detector. The most commercially know device that uses this technology is Kinect 2 [38,39], which provides depth map of 512 × 424 pixels at 30 frames per second.
3. Structured light sensors [40] such as Kinect 1 [41] which was released in November 2010 by Microsoft. Kinect 1 consists of an RGB camera and a depth sensor. The depth sensor provides depth map of 320 × 240 pixels at 30 frames per second.

Similar to ToF depth cameras, action can be encoded in a laser pulse, which is captured by single-pixel cameras. The contents of the scene are encoded in time-series data. When using single pixel camera setups, processing steps such as pose estimation is not necessary. The acquired time series data are usable for machine learning tasks without any modification or additional processing.

### 2.2. Sing-Pixel Single-Photon Acquisition

Recent advances in photonics offer various innovative approaches for three-dimensional imaging [42]. Among those is time-of-flight imaging, which enables detection and tracking of objects. This involves illuminating the scene with diverging light pulses shorter than 100 ps. The light is scattered back from the scene and its flight time is detected with respective accuracy. Flight time *t* of light multiplied by the speed *c* of light directly gives the distance the light pulse has travelled from the source to the detector. Often, the laser source and detector are nearby and the value ct equals twice the distance of the object. Compared to time-of-flight ranging used in LiDARs (Light Detection And Ranging device), the principles introduced here utilise the knowledge of light propagation and are potentially capable of achieving higher spatial resolution.

In early experiments, 50 fs pulse duration mode-locked Ti:Sapphire near-infrared (NIR) laser and streak camera of 15 ps temporal resolution with array matrix were used to detect movement in occluded environment or to recover the 3D shape of an object behind direct line of sight [28,43]. (Using light pulses as short as 50 fs was not necessary; this is a widely spread ultrashort pulse laser source available in photonics labs.) The reconstruction of the object shape required data traces from various viewing angles and mathematical back-projection. In the scope of current research, the non-line-of-sight illumination can be seen as a method of efficiently diverging the incident laser pulse on the scene. There has been several suggestions to use more widely accessible hardware by replacing expensive and fragile streak camera with single photon avalanche diode (SPAD) [29], or to construct a setup based on modulated laser diodes and single pixel photonic mixer device [44].

In proof-of-principle experiment [45] a single pixel SPAD detector (the actual 32 × 32 pixels were used for statistics and to speed up the measurements, such device was and early prototype at the time) was used and ca. 50 ps temporal resolution was utilised to demonstrate the ability to detect linear movement of a non-line-of-sight object. Again, ultrashort 10 fs pulse duration Ti:Sapphire laser with carrier wavelength in NIR region was used. Instead of recording the shape of the object, the shape of its reflection on a screen (a floor) was recorded and position of the object was derived from geometry. Replacing the detector array by three single-pixel SPAD detectors, real-time movement of an object was traced [46]. In this experiment, pulsed NIR diode was used instead of Ti:Sapphire laser. The integration time for single-photon detector was reduced from approximately 3 s to 1 s. In consequent papers, the table-top scenes are scaled up to detect a human [36,47]. Significance of the solution presented in [36] relies on artificial neural network machine learning algorithms for data analysis instead of deterministic tools used before. As a result, the team led by Daniele Faccio was able to distinguish between several standing position of a human and distinct between three different persons by analysing merely one-dimensional trace of SPAD detector.

### 2.3. Action Recognition

Most action recognition and monitoring systems use images with high enough quality where a person can be identified. When considering commercial applications, such systems invade human privacy. The identification factor can be removed by blurring or obscuring the images, downscaling, using encryption and IT solutions to keep the stored data safe. Nevertheless, at some point data is available in a format where people can be identified and can be mishandled due to breach of security, selling private data for commercial purposes or by request from governmental authorities.

One way of removing the privacy concerns is to use devices which by default use low resolution images, hence eliminating privacy issues at the data acquisition step. For such purpose, researchers are developing methods for action recognition using single pixel and low-resolution cameras. A privacy preserving method was proposed Jia and Radke [48] to track a person and estimate pose of a person using a network of ceiling-mounted time-of-flight sensors. Tao et al. [49] based their solution on a network of ceiling-mounted binary passive infrared sensors to recognise a set of daily activities. Kawashima et al. [50] used extremely low-resolution (16 × 16 pixels) infrared sensors to monitor a person constantly day and night without privacy concerns. Ji Dai et al. [51] studied the privacy implications using virtual space for action recognition. They studied Kinect 2 resolutions from 100 × 100 pixels down to 1 × 1 and their effect on action recognition methods. To address privacy issues, Xu et al. [52] proposed a fully-coupled two-stream spatiotemporal architecture for reliable human action recognition on extremely low resolution (e.g., 12 × 16 pixel) videos.

In this research work, we develop a new methodology for action recognition without using any data which can rise a privacy issue. Such a system can be highly used in places such as nursery and hospitals where recognition of actions might be important without violating the privacy rights of people in the environment.

### 2.4. Data Interpretability

In comparison to devices such as Kinect, the depth map provides enough information about a person’s body shape and height, and facial features to visually identify the person and his/her actions in the scene. In the proposed experimental setup, we recorded a kind of a depth map, but it was recorded with a single pixel detector. Hence, the trace has no spatial resolution, which would enable identifying a person or an object directly through detecting above mentioned properties. The spatial properties of the scene are imprinted into the temporal evolution of the recorded trace. In the case an action takes place, characteristic temporal evolution pattern is imprinted to the recorded trace. The recorded 1D time series containing temporal evolution of back scattered light (timestamped detected photon amplitudes) is enough to recognise human actions when interpreted using machine learning algorithms. In the case of a static scene, there is no change in the consequent temporal traces, indicating that no actions are taking place. In addition, the data footprint of a 1D data trace is smaller than that of a depth map. This enables rapid processing times. In the case of using Kinect, the data processing pipeline contains human interpretable data that could be used for unlawful purposes, but in the proposed setup such possibility does not exist.

## 3. Collected Data

**Figure 1:**
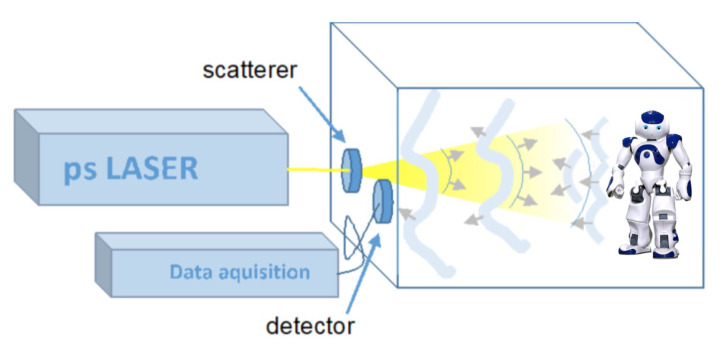
The data collection setup: Fianium laser delivers 30 ps duration light pulses. The collimated laser beam is directed to a scatterer, which creates divergent speckle pattern (giving divergence of 40 degree apex angle) inside the box, which are directed to the black box specially designed for the robot. Scattering illumination will reduce potential interference effects at the detector and, using controlled speckle pattern could be used to increase the lateral resolution. The light scattered from the moving object (NAO V4) and the walls is detected using single-pixel hybrid photodetector (HPD), which detects the temporal evolution of back scattered light.

In this research, for data collection, we created a special setup. Figure 1 shows the general data collection setup, including the placement of the laser and the detector sensor. The data has been collected under the control environment where a NAO V4 humanoid robot was placed in a black box with dimensions of 800 × 800 × 1200 ${\mathrm{mm}}^{3}$ (W × H × L) and was used to conduct some pre-defined actions. The scene was illuminated by Fianium supecontinuum laser source (SC400-2-PP) working at 1 MHz rate. The scatterer ensured that the whole scene Was illuminated at once, without any scanning or other moving parts required. The reflected light from the scene was collected by a Hamamatsu R10467U-06 hybrid photodetector (HPD) with spectral sensitivity range of 220–650 nm. The neutral density filter (OD2) was used in front of the detector to prevent HPD damage due to overexposure. The signal from the HPD was boosted by a Hamamatsu C10778 preamplifier (37 dB, inverting) and then directly digitised by LeCroy WaveRunner 6100a (1 GHz, 10 Gs/s) oscilloscope. The HPD was used in a pulse current (multiphoton) mode, therefore the time resolution of the system was determined by its single-photon pulse response of 600 ps FWHM. The oscilloscope worked in a sequence acquisition mode, recording 200 traces from subsequent trigger events during one sequence, with average frame rate of five sequences per second. The traces within one sequence were averaged to improve signal-to-noise ratio due to both electronic noise and photon statistics. The usage of multiphoton detection mode allowed greatly reducing acquisition time per frame, although with a lower time resolution, unlike the single photon detection used in [46]. The oscilloscope traces in the form of reflected light intensity versus time in nanosecond scale contained all the relevant information about the scene in a non-human-readable form, thus preserving privacy. The series of such traces recorded a 5 fps therefore contain the information about motion.

Various experiments were performed using one- and two-robot setups. A short summary can be seen in Table 1, More detailed description of the tasks can be found in the following sections.

### 3.1. ONE-Robot Setup

**Figure 2:**
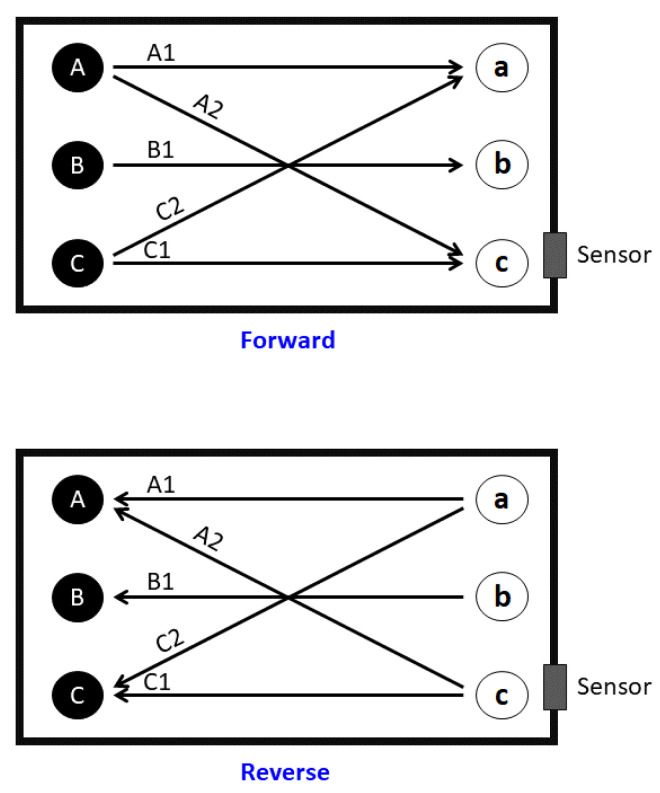
Start and endpoints, showing paths of the robot during directional walk.

**Figure 3:**
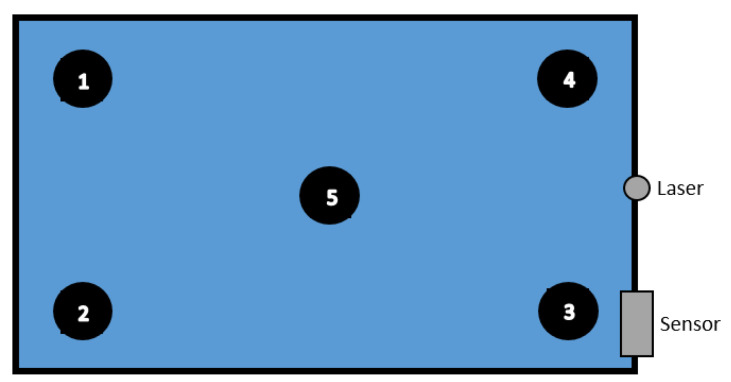
Positions of sitting down and standing up actions.

**Figure 4:**
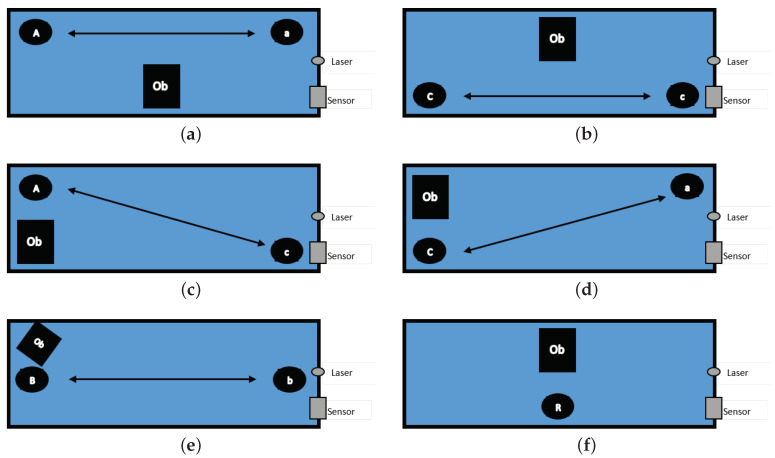
Positions of object during various robot actions.

Initially, for acquiring training data, only one robot was used. Experiments were divided into the following categories:

1. **Directional walk**: We specified three starting points (A, B, and C) and three end points (a, b, and c) inside the box. The robot walked from starting points at 70 cm distance to corresponding end points, and vice versa. In addition, two diagonal directions, from Point A to Point c and from Point C to Point a, were travelled both forward and reverse, as illustrated in Figure 2. All action were repeated 25 times for each point per each direction. These walking actions are shown in Table 2 and Table 3.
2. **Sitting down (sd) from standing up pose and Standing up (su) from sitting down pose**: We specified five areas where the robot was located, as illustrated in Figure 3. These actions were repeated 10 times per area and in each repetition, the position of the robot was nearly the same. Summary of performed tasks is shown in Table 4.
3. **Waving right hand (hw) for 3 s**: This action was repeated 25 times in Areas 2 and 5 (see Table 4).
4. **Include both object and robot**: An object was placed in the environment while the robot was doing the six tasks, which are listed in Table 5 (see Figure 4). Each task was repeated 12 times.

### 3.2. Two-Robot Setup

**Figure 5:**
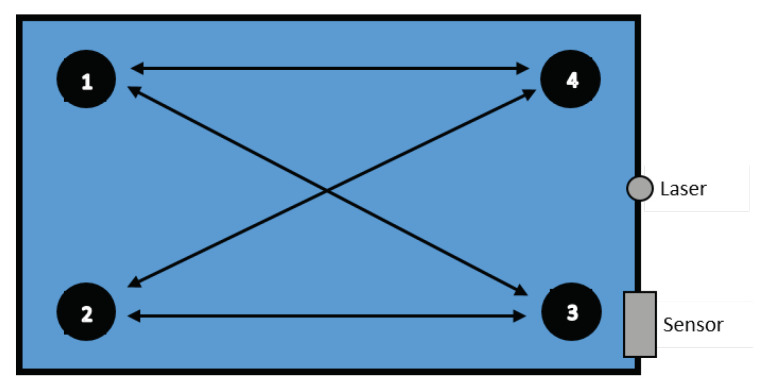
Position of two robots during actions.

We also devised new setup with two NAO V4 humanoid robots. Firstly, one robot was standing still at Position 1 and the other robot at Position 2 walks forward and reverse to Positions 3 and 4, as shown in Figure 5. This action was repeated 10 times. In the next experiment, both robots performed actions simultaneously. Performed actions are listed in Table 6).

**Figure 6:**
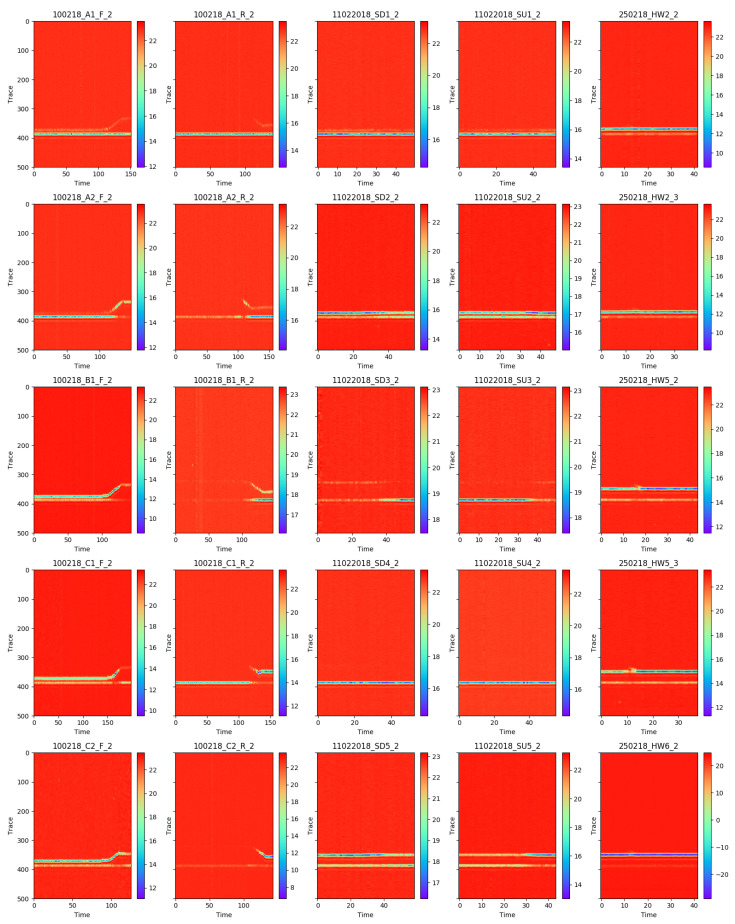
Visualisation of the traces throughout time (x-axis). Columns correspond to different actions (respectively, forward walking, reverse walking, sitting down, standing up, and waving), whereas rows correspond to different examples. The titles on the subplots correspond to the sequence files in the dataset.

In Figure 6, we illustrate a few examples of preprocessed data, which was used in training. Columns correspond to different actions and the rows are different examples. That is, the sequences consisted of a time series of 500-dimensional vectors.

## 4. Method

We chose a recurrent neural network (RNN) as our baseline. Recurrent nets are able to model multivariate time-series—in our case, time-of-flight measurements—and output a class prediction by considering the whole temporal sequence. In particular, our choice was a RNN with Gated-Recurrent Unit (GRU) cells. These cells can retain long-temporal information using internal gates and a set of optimisable parameters.

### 4.1. Gated-Recurrent Unit

We briefly introduce GRUs following the notation from [53]. Let $x=\left(x_{1},\ldots ,x_{t},\ldots ,x_{T}\right),\;x_{t}\in R^{n}$ be a sequence of *T* observations and y∈C its ground truth class label. At each time step *t*, a GRU cell receives $x_{t}$ and outputs an activation $h_{t}\in R^{m}$ response

(1) $$h_{t}^{j}=\left(1-z_{t}^{j}\right)h_{t-1}^{j}+z_{t}^{j}{\tilde{h}}_{t}^{j}$$

by combining activation at previous time step $h_{t-1}^{j}$ and a candidate activation from the current time step ${\tilde{h}}_{t}^{j}.$

The trade-off factor $z_{t}^{j}$, namely *update gate*, is calculated as

(2) $$z_{t}^{j}=\sigma {\left(W_{z}x_{t}+U_{z}h_{t-1}\right)}^{j},$$

where $W_{z}\in R^{m\times n}$ and $U_{z}\in R^{m\times m}$ are optimisable parameters shared across all *t* and σ a sigmoid function that outputs values in the interval (0,1).

In its turn, the *candidate activation* is calculated

(3) $${\tilde{h}}_{t}^{j}=\tanh{\left(Wx_{t}+U\left(r_{t}⊙h_{t-1}\right)\right)}^{j},$$

where ⊙ is the element-wise product of two vectors and $r_{t}$ also known as *reset gate*. Note that $W\in R^{m\times n}$ and $U\in R^{m\times m}$ are different sets of parameters from $W_{z}$ and $U_{z}$.

Similar to the update gate $z_{t}$, the *reset gate* is

(4) $$r_{t}^{j}=\sigma {\left(W_{r}x_{t}+U_{r}h_{t-1}\right)}^{j}.$$

Finally, the last GRU activation at time *T* is input to a dense layer with softmax activation function. From the dense layer, the logit value $z^{i}$ is computed by

(5) $$z^{i}=\sum_{j}w_{s}^{ij}h_{T}^{j},$$

where $W_{s}=\left(w_{s}^{ij}\right)$ are the softmax layer weights. Then, the softmax activation function can be applied to output the sequence classification label

(6) $${\hat{y}}^{i}=\frac{e^{z^{i}}}{\sum_{i}e^{z_{i}}}.$$

### 4.2. Bidirectional GRU and Stacked Layers

Bidirectional recurrent networks consist of two independent networks processing the temporal information in the two temporal dimensions, forward and reverse, so their activation outputs are concatenated. The input of the reverse recurrent network is simply the reversed input sequence. The logit value computation becomes

(7) $$z^{i}=\sum_{j}w_{s}^{ij}\left[h_{\mathrm{fw},T}^{j},h_{\mathrm{rv},0}^{j}\right],$$

where [·,·] is the concatenation of forward and reverse GRUs activations.

In addition, GRU layers can be stacked to form a deeper GRU architecture. The first GRU layer receives as input the sequence of observations x, whereas each subsequent layers are fed with activation outputs from the previous layer. We finally apply the softmax dense layer to the activations of the deepest stacked layer.

### 4.3. Baseline

**Figure 7:**
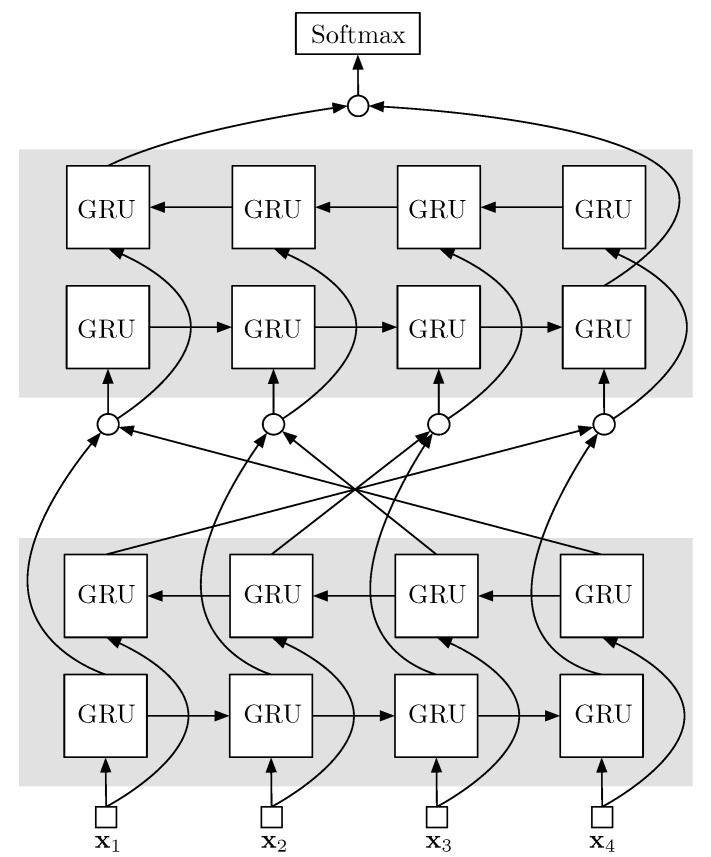
The two-layer bidirectional GRU baseline architecture. Arrays represent information flow, grey rectangles are bidirectional GRU layers, and circles represent the concatenation operation.

Our architecture is a two-layer bidirectional GRU, each GRU with 512 neurons (experimentally chosen). The size of the softmax dense layer is the number of classes |C|. Figure 7 illustrates the architecture.

## 5. Experimental Results and Discussion

### 5.1. Learning Model Details and Code Implementation

Among different RNN cells, we chose Gated-Recurrent Units (GRU) for our baseline architecture. Compared to other recurrent cells, such as Long-Term Short Memory (LSTM) cells, these require a reduced number of parameters while still retaining long-term temporal information and providing highly competitive performance [54]. GRU is also often chosen over LSTM because hidden states are fully exposed and hence easier to interpret.

For the model computations, we entirely relied on GPU programming. In particular, our implementation is based on Keras [55], a GPU-capable deep-learning library written in Python. As for the GPU device itself, we utilised an NVIDIA Titan Xp with 12 GB of GDDR5X memory.

### 5.2. Ablation Experiments on GRU Architectures

To determine the best GRU architecture, we first performed a set of binary classification experiments on the following actions: forward (walking), reverse (walking), sit-down, standing up, and handwaving. We report the performance in terms of accuracy (averaging accuracies over a 10-fold cross validation). In Table 7, we illustrate the ablation experiments on different multi-layer and bidirectional GRU architectures with fixed hidden layer size to 64 neurons. For each architecture and target action, we trained a different GRU model for 25 epochs, which was enough to avoid under-fitting in the most complex model (two-layer biGRU).

In particular, the most complex model, two-layer biGRU, was the one that provided the best result. This showed how both multiple and bidirectional layers can help to model single-pixel time-of-light data sequences. In particular, adding a second stacked layer provided a +5.09% improvement over one single layer, whereas the bidirectionality increased accuracy by 4.4%. The +8.37% gain from using both showed how those two architecture variations are highly complementary when dealing with our data.

Next, using a two-layer biGRU, we performed another set of ablation experiments on hidden layer sizes: {32,64,128,256,512}. Since the hidden layer size drastically affects the number of parameters to optimise during the training stage, each model was trained during a different number of epochs: {10,25,50,100,200,400}, respectively. Results are shown in Table 8.

The largest model, i.e., 512 hidden layer neurons, performed the best. Its +5.62% gain with respect to the smallest two-layer biGRU model with 32 neurons demonstrated room for improvement from using more complex models despite the presumed simplicity of single-pixel time-of-flight time-series. However, we discarded further increasing the hidden size because of computational constraints: enlarging the hidden layer causes an exponential grow of the number of parameters to train. In particular, a model with 32 hidden neurons consisted of 121 K parameters, whereas 512 hidden neurons increased the size up to 7.8 M (and 37.7 M in the case of 1024 neurons); this and the saturation of accuracy discouraged us to keep enlarging the hidden layer size.

Before further experimentation with GRU recurrent nets, we compared the best performing model to its analogous LSTM variant (two-layer biLSTM with 512 hidden layer neurons). In Table 9, we show how GRU could obtain competitive performance with LSTM. The marginal improvement of 0.56% obtained by LSTM requires a substantial increment of the number of parameters, especially when considering larger models. In the case of 512 hidden size, LSTM has 2.6M additional parameters to optimise when compared to the GRU version. For further experiments, we stuck to the biGRU (two-layer, 512 hidden neurons) architecture.

### 5.3. Final Experiments

After having fixed the final GRU model architecture to tow bidirectional stacked layers with 512 hidden neurons, we performed evaluated its performance in multiclass classification and also other experiments to ensure the generalisation capabilities of our approach.

#### 5.3.1. Multiclass Classification

**Figure 8:**
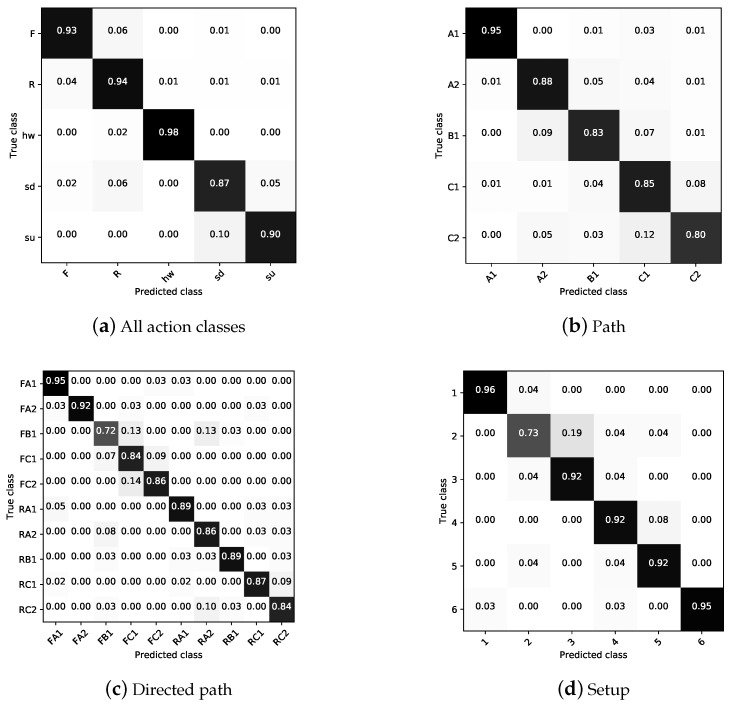
Confusion matrices (row-wise normalised) from multiclass classification experiments from Table 10.

To evaluate the missclassifications and potential confusion among classes from our previous binary problems, we first defined a multiclass problem with labels those same labels: {F, R, sd, su, hw}, where F is forward, R is reverse, sd is sit down, su is stand up, and hw is hand-wave. In this five-class problem, the model was able to correctly predict 92.67% of actions (see column “Actions” in Table 10). As shown in Figure 8a, the confusion is introduced by the semantically similar classes, either forward and reverse or sit-down and stand-up. The hand-wave classification was almost perfect, only confused once as a reverse instance in 50 hw examples.

The second and third experiments were intended to classify the walking path. The former was not distinguishing walking direction. We hence defined two separate sets of labels, {A1, A2, B1, C1, C2} and {FA1, FA2, FB1, FC1, FC2, RA1, RA2, RB1, RC1, RC2}, respectively, where letters (F) and (R) before action label are used to distinguish between action in forward or reverse direction. As shown in Table 10, the model performed similarly in the two cases, with slightly worse performance not considering the walking direction (86.23%) than when doing so (86.65%). Figure 8 shows the confusion matrices for these two experiments.

Finally, in the fourth and last experiment, we labelled the setup in which the action was occurring with labels {1,…,6}, which correspond to tasks listed in Table 5. In this experiment, the robot had to perform various tasks, in addition to performing an action an object is also present in the same environment. Its location and performed actions can be seen in Figure 4. The accuracies obtained from those are summarised in Table 10, while Figure 8 illustrates class confusions.

#### 5.3.2. Model Generalisation on Actions and Two Robots

In this section, we evaluate the generalisation capabilities of the models when learning from single-pixel time-of-flight patterns.

Each action was captured a certain amount of repetitions. During this repetition, the path in walking actions (forward and reverse) or initial position (sit-down, stand-up, and hand-wave) were varied. In this experiment, we wanted to take this into account and try to learn by excluding from training the all the repetitions of one action to assert the model is not overfitting due to repetitions being very similar patterns. For that, we changed our validation procedure to leave-one-rep set-out, i.e., we predicted a repetition set all at once in the test set, and did not use repetitions from the same respect during training. Results are presented in Table 11. If we compare to those to results from the same model, i.e., biGRU (two-layer, 512-hidden), in Table 10, we can observe there was no drop in accuracy, but a slight improvement—probably due to both the generalisation capabilities and the fact that we could use more data to train across folds.

All sequences were with just one robot performing actions. A separate set of sequences was used to test action classification when two robots were present, as shown in Table 12. These sequences were only used in the test phase (only one-robot sequences were used for training). In particular, we analysed three different scenarios: (1) one robot acted, while the other one stood still; (2) the two robots performed the same action; and (3) each robot performed a different action.

From results in Scenario (1), we observed the standing-up robot did not interfere in the other action category prediction. In fact, the model failed to predict stand-up action since the other actions presented a more dominant motion pattern that interfere in the stand-up pattern learned from one-robot actions.

### 5.4. Discussion

In this paper, we propose a concept for detection actions while preserving the test subjects (NAO V4 robot) privacy. Our concept relies on recording only the temporal evolution of light pulses scattered back from the scene. Such data trace to record one action contains sequence of one-dimensional arrays of voltage values acquired by the single-pixel detector after amplifying and detection by the data acquisition system at 6 GHz repetition rate. The data trace is very compact and easy to process, compared to videos, containing sequences of 2D images.

The data volume reduction is achieved by controlled illumination and single pixel detector without any spatial resolution. The scene was illuminated with a diverging, speckled light pulse of 30 picosecond ($30\times 10^{-12}$ ps) duration. The method would also work in different scenes, where most of the objects are static.

Compared to 2D images, hardly any information about the colours, object, their shapes and positions could be retrieved from the data traces by classical method. Although quite similar to the neural networks, a human can distinguish the actions and perhaps also clearly differentiate moving directions from the data traces.

The research in hand clearly articulates the core properties of movement—it imprints a temporal evolution to even most simple data trace. Owing to the interdisciplinary approach through combining the tools of photonics (modern, application oriented optics and light detection) and computer science, one is capable of reducing the data rate. The result has high potential to provide cost effective surveillance systems to aid societies to look after of public order, and take care of young, elderly and injured members.

The photonics and data acquisition schemes used in this experiment are unlikely to become widespread owing to their high cost and other features. However, detectors and laser systems capable of providing suitable illumination and detection properties in affordable price range are being developed and will enter the market in near future.

## 6. Conclusions

This research work proposed a new methodology for action recognition while preserving the test subjects privacy. The proposed method uses only the temporal evolution of light pulses scattered back from the scene. Advanced machine learning algorithms, namely RNN and LSTM, were adopted for data analysis and demonstrated successful action recognition. The experimental results show that our proposed method could achieve high recognition rate for five actions, namely walking forward, walking reverse, sitting down, standing up, and waving hand, with an average recognition rate of 96.47%. In this work, we additionally studied action recognition when multiple concurrent actors are present in the scene.

In future work, we will conduct further experiments, including more complex actions, such as running, jumping, and head movements. We are planning to record higher number of samples to conduct a better generalisation capabilities of our proposed approach. 

## Figures and Tables

**Table 1:** Summary of the performed actions.

	One-Robot						Two-Robot	
Task	Walk Forward	Walk Reverse	Sit Down	Stand Up	Hand Wave	Object Setup	Same Action	Different Actions
Repetitions	125	125	50	50	50	156	70	20

**Table 2:** Forward (F) movement.

Task	A1	A2	B1	C1	C2
Repetitions	25	25	25	25	25
Start location	A	A	B	C	C
Stop location	a	c	b	c	a

**Table 3:** Reverse (R) movement.

Task	A1	A2	B1	C1	C2
Repetitions	25	25	25	25	25
Start location	a	c	b	c	a
Stop location	A	A	B	C	C

**Table 4:** Tasks performed in specific locations.

Task	Sit Down					Stand Up					Hand-Wave	
Repetitions	10	10	10	10	10	10	10	10	10	10	25	25
Location	1	2	3	4	5	1	2	3	4	5	2	5
Action	sd	sd	sd	sd	sd	su	su	su	su	su	hw	hw

**Table 5:** Tasks in presence of an object.

Task	1	2	3	4	5	6 *
Object location	Figure 4a	Figure 4b	Figure 4c	Figure 4d	Figure 4e	Figure 4f
**Walk Forward**	A to a	C to c	A to c	C to a	B to b	hw
Repetitions	12	12	12	12	12	12
**Walk Reverse**	a to A	c to C	a to C	c to A	b to B	su/sd
Repetitions	12	12	12	12	12	12/12

* In Task 6, the robot did not go forward or reverse, but performed hand-wave, stand-up and sit down action, where each action was repeated 12 times.

**Table 6:** Actions performed by two robots.

Repetition	Robot 1 Action	Position	Robot 2 Action	Position
10	Forward	1 to 4	Forward	2 to 3
10	Sit Down	1	Sit Down	2
10	Stand Up	1	Stand Up	2
10	Sit Down	3	Sit Down	4
10	Stand Up	3	Stand Up	4
10	Hand-Wave	1	Hand-Wave	2
10	Hand-Wave	3	Hand-Wave	4
10	Stand	1	Forward	2 to 3
10	Stand	1	Forward	2 to 4

**Table 7:** Comparison on GRU models with multiple layers and/or bidirectionality. In this ablation, we defined a set of five binary problems: forward, reverse, sit-down, stand-up, and hand-wave actions. The results reported are class-weighted accuracies averaged over a 10-fold cross validation. The “Average” column is the average of performances on binary problems.

	Forward	Reverse	Sit-Down	Stand-Up	Handwave	Average
GRU (1-layer, 64-hidden)	87.28	83.85	76.48	78.15	0.945	84.05
GRU (two-layer, 64-hidden)	88.48	90.06	85.75	86.41	94.99	89.14
biGRU (1-layer, 64-hidden)	89.28	87.62	82.49	86.85	96.02	88.45
biGRU (two-layer, 64-hidden)	91.42	91.08	90.07	92.51	97.01	92.42

**Table 8:** Hidden layer size experiments on five binary problems (see Columns 2–6). The results reported are class-weighted accuracies averaged over a 10-fold cross validation. The “Average” column is the average of performances on binary problems.

	Forward	Reverse	Sit-Down	Stand-Up	Handwave	Average
biGRU (two-layer, 32-hidden)	89.97	89.78	87.89	89.47	95.92	90.61
biGRU (two-layer, 64-hidden)	91.42	91.08	90.07	92.51	97.01	92.42
biGRU (two-layer, 128-hidden)	93.10	93.63	92.98	95.32	97.70	94.55
biGRU (two-layer, 256-hidden)	93.76	94.17	94.34	95.52	98.89	95.34
biGRU (two-layer, 512-hidden)	94.94	95.20	95.02	96.70	99.29	96.23

**Table 9:** GRU versus LSTM on 5 binary problems (see Columns 2–6). The results reported are class-weighted accuracies averaged over a 10-fold cross validation. The “Average” column is the average of performances on binary problems.

	Forward	Reverse	Sit-Down	Stand-Up	Handwave	Average
biGRU (two-layer, 64-hidden)	91.42	91.08	90.07	92.51	97.01	92.42
biLSTM (two-layer, 64-hidden)	91.56	96.91	92.02	89.84	94.58	92.98

**Table 10:** Classification on four multiclass problems obtained by biGRU (two-layer, 512-hidden) baseline. The results reported are class-weighted accuracies averaged over a 10-fold cross validation.

Actions	Path	Directed-Path	Setup
92.67	86.23	86.65	90.00

**Table 11:** Leave-one-rep set-out cross-validation (LOROCV) experiment using biGRU (two-layer, 512-hidden). These are the same as those from last row in Table 8, but using LOROCV instead of 10-fold CV.

	F	R	sd	su	hw	Average
biGRU (two-layer, 512-hidden, 10fCV)	94.94	95.20	95.02	96.70	99.29	96.23
biGRU (two-layer, 512-hidden, LOROCV)	96.77	95.14	93.36	97.11	100.0	96.47

**Table 12:** Two-robot experiments in three different scenarios: one robot standing up while other performing a particular action, the two robots performing the same action, and the two performing each a different action. Each scenario is a separate test set with a different number of examples. In brackets, the number of positive examples for each class in each scenario. Since positive/negative classes are, we report class-weighted accuracies (%).

	#{Examples}	F	R	sd	su	hw
One robot standing up and sitting down	100	80.00	88.00	95.00	15.00	100.00
		(50)	(50)	(0)	(100)	(0)
Same two actions	70	25.00	72.86	75.00	54.00	50.00
		(10)	(10)	(20)	(20)	(20)
Two different actions	20	50.00	100.00	95.00	55.00	55.00
		(10)	(0)	(10)	(10)	(10)
